# A Possible Role for Metallic Ions in the Carbohydrate Cluster Recognition Displayed by a Lewis Y Specific Antibody

**DOI:** 10.1371/journal.pone.0007777

**Published:** 2009-11-10

**Authors:** William Farrugia, Andrew M. Scott, Paul A. Ramsland

**Affiliations:** 1 Centre for Immunology, Burnet Institute, Melbourne, Victoria, Australia; 2 Tumour Targeting Program, Ludwig Institute for Cancer Research, Austin Health, Heidelberg, Victoria, Australia; 3 Department of Surgery (Austin Health/Northern Heath), The University of Melbourne, Heidelberg, Victoria, Australia; 4 Department of Immunology, Monash University, Alfred Medical Research and Education Precinct, Melbourne, Victoria, Australia; University of Queensland, Australia

## Abstract

**Background:**

Lewis Y (Le^y^) is a blood group-related carbohydrate that is expressed at high surface densities on the majority of epithelial carcinomas and is a promising target for antibody-based immunotherapy. A humanized Le^y^-specific antibody (hu3S193) has shown encouraging safety, pharmacokinetic and tumor-targeting properties in recently completed Phase I clinical trials.

**Methodology/Principal Findings:**

We report the three-dimensional structures for both the free (unliganded) and bound (Le^y^ tetrasaccharide) hu3S193 Fab from the same crystal grown in the presence of divalent zinc ions. There is no evidence of significant conformational changes occurring in either the Le^y^ carbohydrate antigen or the hu3S193 binding site, which suggests a rigid fit binding mechanism. In the crystal, the hu3S193 Fab molecules are coordinated at their protein-protein interface by two zinc ions and in solution aggregation of Fab can be initiated by zinc, but not magnesium ions. Dynamic light scattering revealed that zinc ions could initiate a sharp transition from hu3S193 Fab monomers to large multimeric aggregates in solution.

**Conclusions/Significance:**

Zinc ions can mediate interactions between hu3S193 Fab in crystals and in solution. Whether metallic ion mediated aggregation of antibody occurs *in vivo* is not known, but the present results suggest that similar clustering mechanisms could occur when hu3S193 binds to Le^y^ on cells, particularly given the high surface densities of antigen on the target tumor cells.

## Introduction

Recent studies of the normal biological functions of the Lewis Y (Le^y^ or CD174) carbohydrate antigen have revealed new insights into its role in cellular function [1]–[3]. This type 2 histo-blood group related carbohydrate antigen is expressed at high surface densities on 60% to 90% of carcinomas of the breast, ovary, colon, lung and prostate [4]–[6]. Together with its frequent over-expression on primary and metastatic tumors, its low abundance and restricted distribution on normal tissues, Le^y^ represents a promising target for antibody-based immunotherapeutic approaches [7], [8].

During human development, Le^y^ is expressed on tissues of the fetus, placenta [9] and newborn [10], [11]. However, in adults Le^y^ is either intracellular or at low surface densities on a few tissues including: hematopoietic precursors, vascular endothelial cells, and epithelial surfaces of the gastrointestinal tract [7], [12]–[14]. Fucosylated type 2 determinants (Le^x^ and Le^y^) have also been demonstrated as the major free oligosaccharides in human seminal plasma [15]. Recently, N-linked Le^y^ oligosaccharides have been shown to be present at high levels in the acrosome (a large intracellular compartment similar to a lysosome) of human sperm, but are not present on the plasma membrane [3]. Defective or malformed sperm were shown to display Le^y^ on the plasma membrane. Given that both Le^x^ and Le^y^ have been shown to interact with human dendritic cells via DC-SIGN to induce T-cell tolerance [16], these oligosaccharides may play a role in the immune privilege of the male reproductive tract [3]. Similarly, tumors may promote T-cell tolerance by expressing high surface levels of type 2 Lewis antigens including Le^y^. More recently, the low level expression of Le^y^ on ICAM-2 of human vascular endothelial cells has been shown to support adhesion and rolling of immature dendritic cells and is involved in the initial cell-cell contacts during angiogenesis [1], [2]. The involvement of Le^y^ in cell adhesion and angiogenic events, together with the high surface densities on Le^y^-positive cancers, suggest the involvement of this carbohydrate antigen in tumor migration (ie., metastasis) and neoangiogenesis [2]. A corollary of these observations is that the mechanism of action of a Le^y^-specific therapeutic antibody may not solely be through antibody-dependent cellular cytotoxicity (ADCC) and complement-mediated cytotoxicity (CDC), but may additionally involve direct inhibition of tumor cell migration and neoangiogenesis.

Early clinical trials with Le^y^-specific murine monoclonal antibodies and antibody-toxin conjugates were limited by immunogenicity, dose limiting toxicity [17] and unexpected side-effects like vascular leakage syndrome (LMB-1, murine B3 antibody linked to *Pseudomonas* exotoxin) [18]. Phase I trials have now been conducted with Le^y^-specific humanized IgG1 monoclonals, IGN311 [19] and hu3S193 [20], [21], and have shown encouraging safety, pharmacokinetic and tumor targeting properties. Trials have also been conducted with a chimeric BR96-doxorubicin conjugate (SGN-15) in a range of cancer patients with some modest clinical activity, but some immune responses towards the BR96-doxorubicin conjugates were noted [22]. Clinical studies with hu3S193 in a variety of Le^y^-expressing cancer patients have demonstrated that this antibody does not induce human anti-humanized antibody (HAHA) responses, selectively targets and accumulates in Le^y^-expressing tumors at high concentrations, retains immune effector function *in vivo*, and does not show saturable binding to any normal tissue compartment [20], [21]. Against Le^y^-expressing tumor cells, hu3S193 has potent *in vitro* immune effector functions, including complement-dependent cytotoxicity (CDC, IC_50_ = 1.0 µg/ml) and antibody-dependent cellular-cytotoxicity (ADCC, IC_50_ = 0.5 µg/ml) [23]. Furthermore, hu3S193 is rapidly internalized through the lysosomal/endosomal pathway in the Le^y^-expressing MCF-7 tumor cells [24]. The preferential binding of hu3S193 to tumor cells with high surface densities of Le^y^ and lack of binding to normal tissues expressing lower levels of Le^y^ suggests that this antibody is involved in carbohydrate cluster recognition as a first step in tumor cell killing.

Previously, we determined the crystal structure of hu3S193 Fab in complex with Le^y^ at a resolution of 1.9 Å [25]. Since hu3S193 binding of Le^y^ was almost identical to that of the BR96 antibody, we proposed that the antibody response to this tumor-associated antigen was structurally conserved. In a subsequent analysis of all reported free and bound Lewis system carbohydrates (Le^a^, Le^b^, Le^x^ and Le^y^), we confirmed the overall structural similarity and rigid nature of these carbohydrate antigens [26]. Thus, the free Le^y^ conformation closely resembles the biologically active or antibody-bound state. Herein we report three-dimensional structures of both the free (unliganded) and bound (Le^y^ tetrasaccharide) hu3S193 Fab in the same orthorhombic *P*2_1_2_1_2_1_ crystals, which were grown in the presence of divalent zinc ions. Comparison of the free and bound Fabs shows that the binding site of hu3S193 does not undergo significant changes during complex formation. Furthermore, the findings of a zinc-dependent Fab dimer in crystals and aggregation in solution induced by Zn^2+^ ions, may point towards a role of divalent metallic ions in antibody-based carbohydrate clustering.

## Methods

### Preparation and Co-Crystallization of hu3S193 Fab with the Le^y^ Tetrasaccharide

The hu3S193 IgG1(κ) antibody was produced and purified as described [23]. The hu3S193 Fab was obtained by plasmin digestion and purified to homogeneity following protocols reported earlier [25]. Crystals of hu3S193 Fab were produced in 2 µl sitting drops in the presence of Le^y^ by vapor diffusion in a 96-well, round bottom, sitting-drop plate (Corning, Acton, MA, USA) using the Crystal Screen HT kit (Hampton Research, Aliso Viejo, CA, USA). The hu3S193 Fab in 30 mM NaCl, 16 mM Tris-HCl (pH 8.0) was at 7 mg/ml and Le^y^ tetrasaccharide (Sigma, St. Louis, MO, USA) at a fourfold molar excess. Crystals used for data collection were produced under the following condition: 25% (v/v) PEG monomethyl ether 550, 0.1 M MES (pH 6.5) and 0.01 M zinc sulfate.

### Collection of X-ray Diffraction Data and Structure Determination

A single crystal (approximate dimensions of 0.2×0.1×0.1 mm) was transferred to a cryoprotectant solution, comprising the crystallization condition supplemented with 5% (v/v) glycerol. The crystal was mounted in a nylon loop and flash-cooled to 100 K in a N_2_ cryostream (Cryojet, Oxford Instruments, Abington, Oxfordshire, UK). X-ray data were obtained using a MicroMax007/R-Axis IV^++^ rotating anode generator system (Rigaku Americas, Woodlands, TX, USA) operated at 40 kV and 20 mA. The X-rays were focused to 0.3 mm diameter using Osmic Blue confocal optics and diffraction images (Δφ = 0.5°) were captured on an R-Axis IV^++^ detector at a crystal-to-detector distance of 200 mm. Diffraction data were processed using the HKL program suite version 1.97.8 [27].

The two hu3S193 Fab molecules were located by molecular replacement (search model was PDB ID: 1S3K; [25]) using Molrep, version 9.2 [28], as implemented within the CCP4 program suite, version 5.0.5.2 [29]. After rigid body refinement and restrained refinement against a maximum likelihood function (MLF) with Refmac5, the *R_work_* and *R_free_* values were 0.31 and 0.41, respectively. Further fitting of atomic models to electron density maps and crystallographic refinements were performed with TURBO-FRODO, version 5.5, (BioGraphics, Marseille, France) and CNS, version 1.0 [30]. Rigid body refinement of the positions of the VL:VH and CL:CH1 domain pairs, simulated annealing, energy minimization, and temperature factor refinement lowered the *R_work_* to 0.27 and *R_free_* to 0.33. After reiterative fitting into |*F_o_*|−|*F_c_*| electron density maps and crystallographic refinements the final structure contained: a Le^y^ tetrasaccharide associated with Fab1, a glycerol in the binding site of Fab2. In addition, the structure contained 4 Zn^2+^ ions and 143 solvent (water) molecules. The final coordinates had *R_work_* and *R_free_* values of 0.21 and 0.26 at 2.5 Å resolution. A summary of data collection and crystallographic refinement statistics are presented in Table 1. The 1.9 Å resolution hu3S193 Fab-Le^y^ complex (PDB ID: 1S3K) used here for comparison was previously reported [25]. Atomic co-ordinates and structure factors for the current structure have been deposited with the Protein Data Bank at the Research Collaboratory for Structural Bioinformatics http://www.rcsb.org/pdb under the accession code 3EYV.

**Table 1:** Data collection and crystallographic refinement statistics.

Parameter	Value^a^
Data collection	
Space group	*P2_1_2_1_2_1_*
Unit cell variables	
a, b, c (Å)	78.8, 101.5, 115.0
α, β, γ (°)	90, 90, 90
Resolution range (Å)	100–2.50 (2.59–2.5)
Number of unique reflections	31623 (3074)
Percent data completeness	95.8 (94.7)
Average multiplicity	5.5 (5.4)
*R_sym_*	0.073 (0.38)
Mean I/σ(I)	23.8 (4.5)
Crystallographic refinement	
*R_work_*	0.212
*R_free_*	0.263
Ramachandran plot values (%)	
Most favored regions	86.6
Additional allowed regions	12.3
Generously allowed regions	0.8
Disallowed regions	0.3

a Values in parentheses refer to the highest resolution shell, 2.59–2.5 Å, in the data. Refinement and stereochemical parameters were compiled from the CNS program suite, version 1.0 [30] or PROCHECK version 3.3 [59].

### Dynamic Light Scattering (DLS)

The DLS measurements were carried out with a Zetasizer Nano ZS instrument (Malvern Instruments, UK), which was fitted with a 633 nm laser and a single detector located at 173° with respect to the laser. Samples of hu3S193 Fab were prepared in Tris-buffered saline (pH 7.4) and titrated with zinc or magnesium chloride. Immediately before use, samples were centrifuged at 15,850 g at room temperature for 10 min to remove particulate material. The supernates were transferred to plastic cuvettes and allowed to equilibrate at 25 °C for 30 min prior to measurement. The data were analyzed with Malvern Instrument Dispersion Technology Software (DTS), version 3.30. Light scattering intensities (I) were integrated as correlograms (plots of a correlation coefficient, G(τ) = <I(t).I(t+τ)> *versus* time, where τ =  the sampling time of the correlator) and were used to monitor sample dispersity and protein aggregation. The z-average hydrodynamic diameters (*D_H_*) were determined by the cumulants method [31] as defined in the international standard for DLS measurements (ISO 13321).

## Results

### Presence of the Free and Le^y^-Bound hu3S193 Fab Molecules in the Same Crystal

**Figure 1:**
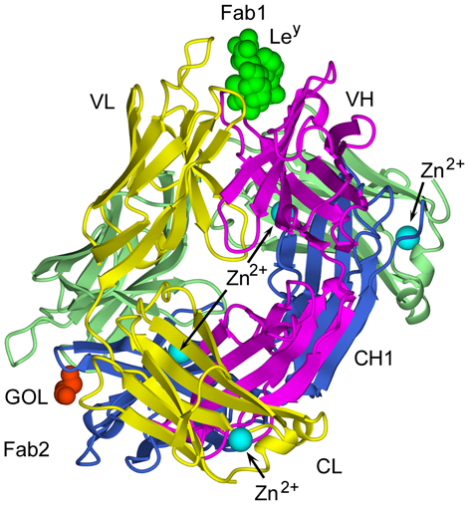
Ribbons style representation of the two hu3S193 Fab molecules comprising the asymmetric unit of the orthorhombic *P*2_1_2_1_2_1_ crystals. Fab1 contains the bound Le^y^ tetrasaccharide (green), while Fab2 contains a loosely bound glycerol (GOL, orange). The light (Fab1, yellow; Fab2, pale green) and heavy (Fab1, magenta; Fab2, dark blue) chains and locations of four divalent zinc ions (cyan) are indicated.

Orthorhombic *P*2_1_2_1_2_1_ crystals of hu3S193 Fab were grown in the presence the Le^y^ tetrasaccharide and the crystallographic structure was determined to 2.5 Å resolution (Table 1). The asymmetric unit contained two hu3S193 Fab molecules (Fig. 1), one binding site was in complex with Le^y^ (Fab1) and the second binding site contained only a loosely associated glycerol molecule and a few solvent molecules (Fab2). Thus, the same crystal contained binding site structures for both the free and Le^y^-bound hu3S193 antibody. Four Zn^2+^ ions were located in the asymmetric unit as strong |*F_o_*|−|*F_c_*| electron density peaks. Two of the Zn^2+^ ions were associated with cross-pairing of the hu3S193 Fab molecules; while the other Zn^2+^ ions were located in solvent-exposed locations at the end of the CL domains (see Fig. 1).

Comparison of the quaternary structures of the originally reported hu3S193 Fab structure (PDB ID: 1S3K) [25] with the two Fab structures reported here revealed very similar structures of the VL:VH and CL:CH1 domain modules, with carbon-*alpha* (Cα) root-mean-square deviations (RMSD) of 0.37–0.40 Å for VL:VH (232 residues) and 0.49–0.80 Å for CL:CH1 (196 residues) in pairwise comparisons. However, the “elbow bend” angles differed between the 1S3K Fab (136°) and the two Fab molecules in the asymmetric unit (Fab1, 143°; Fab2, 146°), indicating a modest degree of flexibility for the hu3S193 Fab in the V-C “switch” regions (*ie.*, short regions of polypeptide connecting the V and C domains). The elbow bend angles of the hu3S139 Fab molecules (136°–146°) are well within the ranges previously observed for Fab and represent frequently occurring values for Fab containing κ-type light chains [32].

### Comparison of the Binding Sites of the Free and Le^y^-Bound hu3S193 Fab Molecules

**Figure 2:**
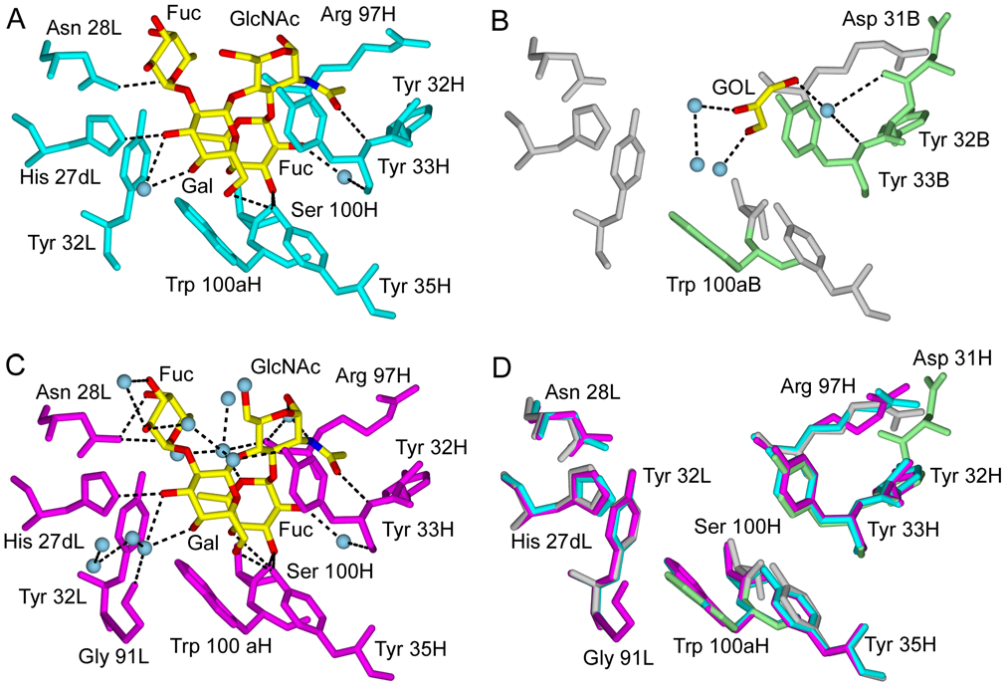
Comparison of the binding sites of three distinct structures of hu3S193 Fab. A) Fab1 containing a bound Le^y^ tetrasaccharide (contact residues in cyan); B) Fab2 containing a loosely bound glycerol and solvent (contact residues highlighted in pale green); C) Structure of hu3S193 Fab in complex with Le^y^ (contact residues in magenta) determined at 1.9 Å resolution (PDB ID: 1S3K) [25]; and, D) Overlays of binding site residues for the three different hu3S193 Fabs. The Le^y^ and GOL ligands are colored by atom type (C, yellow; N, blue; O, red). Ordered solvent molecules participating in the interactions with ligand are shown (panels A–C, pale blue) and hydrogen bonds are drawn as dashed lines (black). Binding site residues are numbered according to the Kabat scheme.

In the two independent hu3S193 Fab-Le^y^ complexes (Fab1 and 1S3K), the interactions between binding site residues and Le^y^ are nearly identical (Fig. 2 A and C). The only notable difference is the absence in the Fab1-Le^y^ complex of a single hydrogen bond between Asn 28L (ND2 atom) and the Le^y^-specific Fuc (O4 atom) saccharide unit (*ie.,* the α1–2 linked Fuc). The Asn 28L to Fuc (ND2 to O4) distance in the Fab1-Le^y^ complex is 3.6 Å, compared to 3.0 Å in the 1S3K complex. Combined with other distances around the Le^y^-specific Fuc, which are consistently slightly larger in the Fab1-Le^y^ complex compared to 1S3K, it appears that Le^y^ is more loosely bound in crystals grown in the presence of zinc ions. This observation is supported by the absence of a Le^y^ ligand in the binding site of Fab2 (Fig. 2 B). The Fab2 binding site is contains a few solvent molecules and a glycerol (GOL) molecule, which is weakly held in place by a single water-mediated hydrogen bond. No binding site residues of Fab2 interacted directly with the GOL molecule, which originated from the cryoprotectant solution used to flash-cool the crystal for X-ray diffraction data collection. The Le^y^ ligand either was not bound or was eluted from Fab2 during transfer to the cryoprotectant, but the Fab2 binding site represents the unliganded or free hu3S193 Fab.

Overlays of the hu3S193 Fab structures (two in complex with Le^y^ and one unliganded Fab) demonstrate almost identical positions for all binding site residues in contact with the Le^y^ tetrasaccharide (Fig. 2 D). Thus, the binding site of hu3193 does not appear to have undergone conformational changes during complex formation with Le^y^. We previously reported that the hu3S193-bound conformation of Le^y^ was fundamentally the same as the known free and bound conformations of Le^y^ and other Lewis system carbohydrates [26]. Similarly, the bound conformations of Le^y^ in 1S3K and Fab1 were essentially the same (Table 2), which further supports the idea of the rigid character of the Le^y^ carbohydrate determinant. Note that the slight variation in the Fucα1–2Gal glycosidic linkage (Table 2) is compatible with the small differences observed in the interactions between the Le^y^-specific Fuc and the hu3S193 binding site for the 1S3K and Fab1 structures (Fig. 2 A and C).

**Table 2:** Conformational characteristics of hu3S193-bound Le^y^ tetrasaccharides.

Fab-Le^y^ complex	Fucα1-2Gal^a^		Galβ1-4GlcNAc		Fucα1-3GlcNAc	
	φ	ψ	φ	ψ	φ	ψ
1S3K	−78.1	140.4	−77.6	139.2	−77.8	−103.4
Fab1	−74.8	132.9	−78.1	134.2	−77.5	−102.5

a The glycosidic dihedral angles φ and ψ (°) are defined as O5−C1−O1−C_x_ and C1−O1−C_x_−C_x−1_, respectively. Values of glycosidic dihedrals for other free and bound Lewis system oligosaccharides were previously reported [26].

At 1.9 Å resolution an extensive solvent network of 13 ordered water molecules surrounds the Le^y^ tetrasaccharide in complex with hu3S193 Fab [25]. The ordered solvent was involved in hydrogen bonding to protein and carbohydrate residues (7 water molecules) and in forming more extended hydrogen bonding networks (6 water molecules), which led to greater complementarity between Le^y^ and the hu3S193 binding site [26]. In the vicinity of the Fab1-Le^y^ interaction, two ordered solvent molecules were observed in the 2.5 Å resolution electron density maps (Fig. 2 A) and both occupied the same locations as in the 1S3K structure (Fig. 2 C). Without the Le^y^ ligand, the hu3S193 binding site contained 4 ordered water molecules and a glycerol. One water molecule in the unliganded hu3S193 binding site occupied a location (near tyrosines 32H and 33H) where the N-acetyl group of the Le^y^ tetrasaccharide binds and could have been displaced during complex formation (Fig. 2 A and B). However, none of the water molecules in the unliganded binding site occupied comparable locations to solvent molecules in the hu3S193 complexes with Le^y^. Thus, it is likely that the water molecules were associated with the Le^y^ carbohydrate or are recruited to the interaction rather than being already present in the hu3S193 binding site.

### Zinc-Dependent Crystallographic Dimers of hu3S193 Fab Suggest a Possible Role of Divalent Metal Ions in Carbohydrate Cluster Recognition

**Figure 3:**
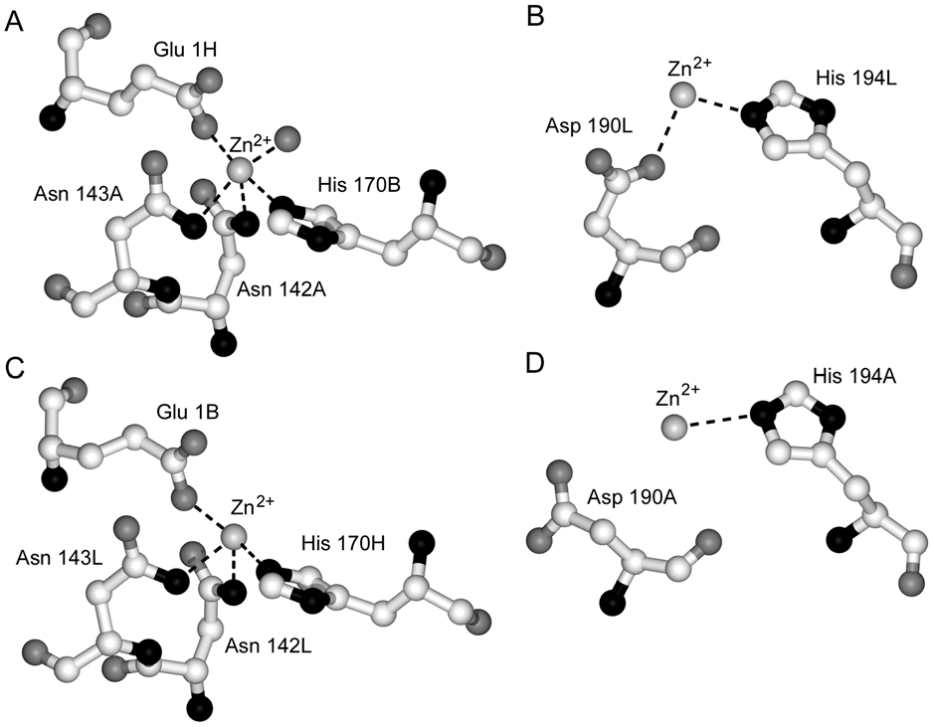
Coordination of divalent zinc ions in orthorhombic crystals of hu3S193 Fab. Two Zn^2+^ ions (A and C) are involved in tetravalent coordination with residues from the two independent crystallographic Fabs. At one site (A) a water molecule interacts with the Zn^2+^ ion, but this interaction does not occur at the second site (C). An additional two Zn^2+^ ions were observed (B and D) and were coordinated by Asp 190L and His 194L (Fab1) or only by His 194A (Fab2) due to small differences in the polypeptide conformation between Fab1 and Fab2.

The hu3S193 Fab molecules contain four Zn^2+^ ions bound in two types of environment. Firstly, protein interface zinc-binding sites are formed between the Fab1 and Fab2 molecules and involve the tetravalent coordination by Fab residues (Fig. 3 A and C). Asparagines 142 and 143 of the light chain (A or L) and His 170 of the heavy chain (B or H) of one Fab form a pocket in the CL:CH1 domain interface that binds a Zn^2+^ ion, which accepts Glu 1 from the heavy chain of the second Fab. In one site, a water molecule acts as a fifth ligand for the Zn^2+^ ion (Fig. 3 A), but this was not observed in the second protein interface zinc-binding site (Fig. 3 C). Access for the water ligand is possible since the coordination of zinc is distorted from tetrahedral geometry. The two zinc ions form part of a larger protein-protein interface between the hu3S193 Fab molecules in the asymmetric unit. Secondly, surface-located zinc sites were identified in the hu3S193 κ-type light chains where Zn^2+^ ions interacted bivalently with His 194L and Asp190L (Fab1; Fig. 3 B) or monovalently with His 194L (Fab2; Fig. 3 D). Rather than being buried at a protein-protein interface the surface-located zinc ions line solvent channels in the orthorhombic *P*2_1_2_1_2_1_ crystals and are at least 4 Å from any symmetry related Fab.

**Figure 4:**
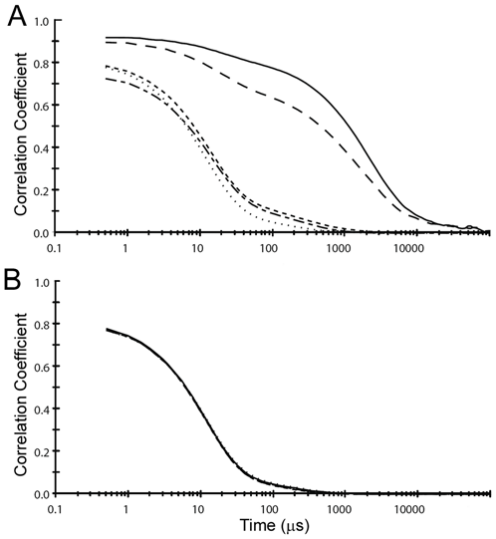
Divalent zinc ion mediated aggregation of the hu3S193 Fab. Dynamic light scattering was used to monitor time-dependent fluctuations (correlograms) in scattered light intensity for samples of hu3S193 Fab (∼15 µM) in the presence of: A) ZnCl_2_; and, B) MgCl_2_. Final concentrations of the divalent metal ions were 0 µM (short dash), 10 µM (dash-dot), 20 µM (dots), 40 µM (dash) and 50 µM (solid).

The presence in crystals of zinc-stabilized hu3S193 Fab homodimers led us to test in solution by DLS the effect of zinc ions on the Fab. In solution, hu3S193 Fab (∼15 µM) aggregated in the presence of 40 and 50 µM ZnCl_2_, but not at lower concentrations or at any concentration of MgCl_2_ tested (Fig. 4). Interestingly, zinc-induced aggregation of the hu3S193 Fab required at least two molar equivalents of Zn^2+^ ions, which is similar to the four Zn^2+^ ions found, associated with the two hu3S193 Fab molecules in the asymmetric unit of the crystals.

**Figure 5:**
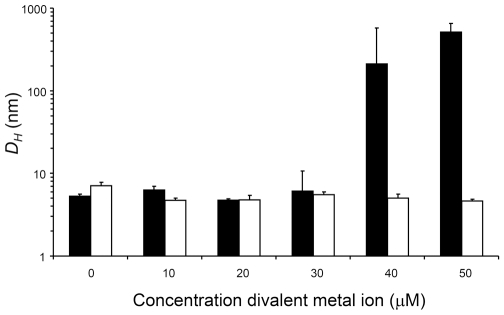
Changes in size of hu3S193 Fab in the presence of zinc ions monitored by DLS. The z-average *D_H_* (nm) was determined for hu3S193 Fab samples in the presence of increasing concentrations of ZnCl_2_ (black) or MgCl_2_ (white). Mean values (n = 3) are shown and error bars represent three standard deviations.

The zinc ion induced changes in solution of hu3S193 Fab were further characterized by DLS by monitoring the z-average hydrodynamic diameters (*D_H_*) in the presence of divalent metallic ions (Fig. 5). Without any metallic ions hu3S193 Fab behaved as a monomer with *D_H_* in the range of 5.3 nm to 7.1 nm. Similar *D_H_* values were obtained at all MgCl_2_ concentrations and when ZnCl_2_ was between 10 µM and 30 µM. A sharp transition occurred at 40 µM ZnCl_2_ where the hu3S193 Fab formed large aggregates with a mean *D_H_* value of 211.9 nm (range of 92.8 nm to 333.9 nm). When the ZnCl_2_ was raised to 50 µM the hu3S193 Fab aggregates had a mean *D_H_* of 511.3 nm (range of 458.5 nm to 547.8 nm). The zinc-mediated aggregates of hu3S193 Fab are substantially larger than polymeric IgM (molecular mass >950 kDa or 19 S), which we have previously shown to have a z-average *D_H_* of between 34 and 37 nm [33], [34]. Thus, in solution zinc ions can mediate the formation of multimeric aggregates of hu3S193 Fab, but does not appear to produce smaller ordered multimers such as the dimers observed in crystals.

While the physiological levels of Zn^2+^ in blood plasma is around 20 µM, certain tissues and cellular compartments have been shown to have dramatically higher levels of Zn^2+^ [35]–[39]. Furthermore, intracellular Zn^2+^ fluxes have been recently shown to be involved in lipopolysaccharide-induced signals in human monocytes [40], which supports the concept that the local concentration of zinc ions can rapidly change in biological microenvironments. Thus, the 40 µM ZnCl_2_ required for aggregation of hu3S193 Fab in solution indicates that *in vivo* aggregation should not occur in the blood plasma, but would possibly be induced in certain tissue or cellular compartments where Zn^2+^ ions can be at significantly higher concentrations.

## Discussion

In the current work, we were presented with an opportunity to examine the three-dimensional structures of both the free and Le^y^-bound hu3S193 binding sites from the same crystal. Apart from the subtle differences in solvent structure and minor adjustments of the Le^y^-specific Fuc residue, the binding sites for the three hu3S193 structures were identical (see Fig. 2 D). Taken together with the established rigid nature of the Le^y^ carbohydrate [26], the available data strongly supports the binding of the Le^y^ antigen by the hu3S193 antibody to resemble the fit of an unbendable key into a rigid lock. This finding is in contrast to the now commonly held view that both antigen and antibody frequently undergo conformational changes or induced fit upon binding [41]–[46]. Similarly, carbohydrates have traditionally been viewed as more flexible and mobile in solution when compared to globular proteins. Thus, the interaction between the hu3S193 antibody and Le^y^ carbohydrate determinant has made us revisit the lock-and-key concept as a possible mechanism for antibody recognition of carbohydrate antigens, particularly in Le^y^-expressing tumors.

Reynolds and colleagues recently examined the hydration features of free type 2 Lewis antigens (Le^x^ and Le^y^) by molecular dynamics (MD) simulations [47]. In this study, the solvated carbohydrate determinants remained relatively inflexible or rigid during extensive MD simulations, confirming earlier observations that the free conformation is representative of the biologically active state for Lewis system antigens (reviewed in [26]). Interestingly, the water molecule bridging events or solvent structure around free Le^y^ observed by the MD simulations were mostly represented by water molecules and a few binding site residues in the hu3S193 Fab-Le^y^ complex at 1.9 Å resolution [25], [47]. For the present complex (Fab1-Le^y^) that was determined at a resolution of 2.5 Å, we identified two of the same bridging water molecules and these were buried in the carbohydrate-antibody interface. Since most of the water molecules surrounding Le^y^ are not buried in the binding site, it is possible that these were simply not observed in crystals at a resolution of 2.5 Å. However, the finding of key water molecules involved in the hu3S193 interaction with Le^y^ supports the conclusion that solvent mediates the rigid conformational properties of this carbohydrate epitope and is important for antibody recognition. Furthermore, the hydrated structure of Le^y^ antigens when presented on the surface of tumor cells is likely to snugly fit the hu3S193 binding site without the need for displacement of the majority of the water molecules from the carbohydrate.

The *in vivo* specificity of hu3S193 for Le^y^ on tumor cells and no saturable binding to any normal tissue compartment [20], indicates that the presentation of Le^y^ determinants by tumor cells is different from normal tissues. Clearly, Le^y^ epitopes are both expressed in tumors at relatively high surface densities and are aberrantly expressed on various membrane-bound glycoproteins including epidermal growth factor receptors, which are also candidate antigens for tumor immunotherapy [48]–[53]. Our findings of a zinc-dependent homodimer of hu3S193 Fab in crystals and the corresponding aggregation of Fab in solution, provides the first evidence for a possible new mechanism of carbohydrate cluster recognition. We propose that binding of hu3S193 to dense clusters of Le^y^ on tumor cells could be further stabilized by Zn^2+^ or other divalent metallic ions resulting in an increase in the avidity of the interaction. Similarly the role of zinc in protein-protein interactions has been described for several other biological systems [54]–[57]. The low surface densities of Le^y^ carbohydrates on normal tissues would not be suitable for lateral zinc-mediated interactions to occur between neighboring antibodies, which could explain the evident lack of binding by hu3S193 to normal Le^y^-expressing tissues.

Another immunological solution for specific antibody binding to dense clusters has been reported for the 2G12 IgG, which is highly specific for the complex oligomannose glycans that decorate the “silent face” HIV-1 gp120 [58]. Both the 2G12 Fab and the intact antibody has been shown to contain domain-swapped Fab homodimers, where VH domains from each Fab associate with the corresponding VL in the adjacent Fab to form an extended surface for multivalent carbohydrate binding. While domain-swapped antibodies represent an elegant mechanism for carbohydrate cluster recognition, these are likely to be rare and difficult to elicit by standard immunization strategies [58]. Our proposed metallic ion mediated mechanism for Le^y^ carbohydrate cluster recognition by hu3S193 may be more general since most IgG/κ antibodies have the residues involved in coordinating Zn^2+^ by hu3S193 Fab.

Understanding the mechanism of action of a candidate therapeutic antibody against solid tumors requires a detailed physicochemical understanding of specificity and immune effector functions as well as the *in vivo* pharmacokinetics and biological activity in normal and tumor sites. We have shown that the binding specificity for Le^y^ by hu3S193 does not involve conformational changes and the interaction mimics the hydration patterns of free Le^y^ antigens. Additionally, the structural results presented here indicate a new potential mechanism for hu3S193 selective recognition of Le^y^ on tumor cells as opposed to normal tissues, which is based on metallic ion mediated carbohydrate cluster recognition.

## References

[1] Garcia-Vallejo JJ, van Liempt E, da Costa Martins P, Beckers C, van het Hof B (2008). DC-SIGN mediates adhesion and rolling of dendritic cells on primary human umbilical vein endothelial cells through LewisY antigen expressed on ICAM-2.. Mol Immunol.

[2] Moehler TM, Sauer S, Witzel M, Andrulis M, Garcia-Vallejo JJ (2008). Involvement of alpha 1-2-fucosyltransferase I (FUT1) and surface-expressed Lewis(y) (CD174) in first endothelial cell-cell contacts during angiogenesis.. J Cell Physiol.

[3] Pang PC, Tissot B, Drobnis EZ, Sutovsky P, Morris HR (2007). Expression of bisecting type and Lewisx/Lewisy terminated N-glycans on human sperm.. J Biol Chem.

[4] Murata K, Egami H, Shibata Y, Sakamoto K, Misumi A (1992). Expression of blood group-related antigens, ABH, Lewis(a), Lewis(b), Lewis(x), Lewis(y), CA19-9, and CSLEX1 in early cancer, intestinal metaplasia, and uninvolved mucosa of the stomach.. Am J Clin Pathol.

[5] Sakamoto J, Furukawa K, Cordon-Cardo C, Yin BW, Rettig WJ (1986). Expression of Lewisa, Lewisb, X, and Y blood group antigens in human colonic tumors and normal tissue and in human tumor-derived cell lines.. Cancer Res.

[6] Yin BW, Finstad CL, Kitamura K, Federici MG, Welshinger M (1996). Serological and immunochemical analysis of Lewis y (Ley) blood group antigen expression in epithelial ovarian cancer.. Int J Cancer.

[7] Kitamura K, Stockert E, Garin-Chesa P, Welt S, Lloyd KO (1994). Specificity analysis of blood group Lewis-y (Le(y)) antibodies generated against synthetic and natural Le(y) determinants.. Proc Natl Acad Sci USA.

[8] Scott AM, Welt S (1997). Antibody-based immunological therapies.. Curr Opin Immunol.

[9] Minas V, Mylonas I, Schiessl B, Mayr D, Schulze S (2007). Expression of the blood-group-related antigens Sialyl Lewis a, Sialyl Lewis x and Lewis y in term placentas of normal, preeclampsia, IUGR- and HELLP-complicated pregnancies.. Histochem Cell Biol.

[10] Heller DS, Thung SN (1990). Expression of Lewis(x) and Lewis(y) blood group related antigens in fetal livers.. Pediatr Pathol.

[11] Candelier JJ, Mollicone R, Mennesson B, Coullin P, Oriol R (2000). Expression of fucosyltransferases in skin, conjunctiva, and cornea during human development.. Histochem Cell Biol.

[12] Mollicone R, Bara J, Le Pendu J, Oriol R (1985). Immunohistologic pattern of type 1 (Lea, Leb) and type 2 (X, Y, H) blood group-related antigens in the human pyloric and duodenal mucosae.. Lab Invest.

[13] Kim YS, Yuan M, Itzkowitz SH, Sun QB, Kaizu T (1986). Expression of LeY and extended LeY blood group-related antigens in human malignant, premalignant, and nonmalignant colonic tissues.. Cancer Res.

[14] Cao Y, Merling A, Karsten U, Schwartz-Albiez R (2001). The fucosylated histo-blood group antigens H type 2 (blood group O, CD173) and Lewis Y (CD174) are expressed on CD34+ hematopoietic progenitors but absent on mature lymphocytes.. Glycobiology.

[15] Chalabi S, Easton RL, Patankar MS, Lattanzio FA, Morrison JC (2002). The expression of free oligosaccharides in human seminal plasma.. J Biol Chem.

[16] van Liempt E, Bank CM, Mehta P, Garcia-Vallejo JJ, Kawar ZS (2006). Specificity of DC-SIGN for mannose- and fucose-containing glycans.. FEBS Lett.

[17] Pai-Scherf LH, Carrasquillo JA, Paik C, Gansow O, Whatley M (2000). Imaging and phase I study of 111In- and 90Y-labeled anti-LewisY monoclonal antibody B3.. Clin Cancer Res.

[18] Pai LH, Wittes R, Setser A, Willingham MC, Pastan I (1996). Treatment of advanced solid tumors with immunotoxin LMB-1: an antibody linked to Pseudomonas exotoxin.. Nat Med.

[19] Szolar OH, Stranner S, Zinoecker I, Mudde GC, Himmler G (2006). Qualification and application of a surface plasmon resonance-based assay for monitoring potential HAHA responses induced after passive administration of a humanized anti Lewis-Y antibody.. J Pharm Biomed Anal.

[20] Scott AM, Tebbutt N, Lee FT, Cavicchiolo T, Liu Z (2007). A phase I biodistribution and pharmacokinetic trial of humanized monoclonal antibody Hu3s193 in patients with advanced epithelial cancers that express the Lewis-Y antigen.. Clin Cancer Res.

[21] Krug LM, Milton DT, Jungbluth AA, Chen LC, Quaia E (2007). Targeting Lewis Y (Le(y)) in small cell lung cancer with a humanized monoclonal antibody, hu3S193: a pilot trial testing two dose levels.. J Thorac Oncol.

[22] Saleh MN, Sugarman S, Murray J, Ostroff JB, Healey D (2000). Phase I trial of the anti-Lewis Y drug immunoconjugate BR96-doxorubicin in patients with lewis Y-expressing epithelial tumors.. J Clin Oncol.

[23] Scott AM, Geleick D, Rubira M, Clarke K, Nice EC (2000). Construction, production, and characterization of humanized anti-Lewis Y monoclonal antibody 3S193 for targeted immunotherapy of solid tumors.. Cancer Res.

[24] Kelly MP, Lee FT, Tahtis K, Smyth FE, Brechbiel MW (2007). Radioimmunotherapy with {alpha}-Particle Emitting 213Bi-C-Functionalized trans-Cyclohexyl-Diethylenetriaminepentaacetic Acid-Humanized 3S193 Is Enhanced by Combination with Paclitaxel Chemotherapy.. Clin Cancer Res.

[25] Ramsland PA, Farrugia W, Bradford TM, Hogarth PM, Scott AM (2004). Structural convergence of antibody binding of carbohydrate determinants in lewis y tumor antigens.. J Mol Biol.

[26] Yuriev E, Farrugia W, Scott AM, Ramsland PA (2005). Three-dimensional structures of carbohydrate determinants of Lewis system antigens: implications for effective antibody targeting of cancer.. Immunol Cell Biol.

[27] Otwinowski Z, Minor W (1997). Processing of X-ray diffraction data collected in oscillation mode.. Meth Enzymol.

[28] Vagin A, Templyakov A (1997). MOLREP: an automated program for molecular replacement.. J Appl Cryst.

[29] Bailey S (1994). The CCP4 suite: programs for protein crystallography.. Acta Crystallogr.

[30] Brunger AT, Adams PD, Clore GM, DeLano WL, Gros P (1998). Crystallography & NMR system: A new software suite for macromolecular structure determination.. Acta Crystallogr.

[31] Koppel DE (1972). Analysis of Macromolecular Polydispersity in Intensity Correlation Spectroscopy: The Method of Cumulants.. J Chem Phys.

[32] Stanfield RL, Zemla A, Wilson IA, Rupp B (2006). Antibody elbow angles are influenced by their light chain class.. J Mol Biol.

[33] Vallas V, Farrugia W, Raison RL, Edmundson AB, Ramsland PA (2007). Dissimilar aggregation processes govern precipitation and gelation of human IgM cryoglobulins.. J Mol Recognit.

[34] Ramsland PA, Terzyan SS, Cloud G, Bourne CR, Farrugia W (2006). Crystal structure of a glycosylated Fab from an IgM cryoglobulin with properties of a natural proteolytic antibody.. Biochem J.

[35] Folin M, Contiero E, Vaselli GM (1994). Zinc content of normal human serum and its correlation with some hematic parameters.. Biometals.

[36] Morrison B, Shenkin A, McLelland A, Robertson DA, Barrowman M (1979). Intra-individual variation in commonly analyzed serum constituents.. Clin Chem.

[37] Kim BJ, Kim YH, Kim S, Kim JW, Koh JY (2000). Zinc as a paracrine effector in pancreatic islet cell death.. Diabetes.

[38] Edstrom AM, Malm J, Frohm B, Martellini JA, Giwercman A (2008). The major bactericidal activity of human seminal plasma is zinc-dependent and derived from fragmentation of the semenogelins.. J Immunol.

[39] Esqueda AC, Lopez JA, Andreu-de-Riquer G, Alvarado-Monzon JC, Ratnakar J (2009). A new gadolinium-based MRI zinc sensor.. J Am Chem Soc.

[40] Haase H, Ober-Blobaum JL, Engelhardt G, Hebel S, Heit A (2008). Zinc signals are essential for lipopolysaccharide-induced signal transduction in monocytes.. J Immunol.

[41] Herron JN, He XM, Ballard DW, Blier PR, Pace PE (1991). An autoantibody to single-stranded DNA: comparison of the three-dimensional structures of the unliganded Fab and a deoxynucleotide-Fab complex.. Proteins.

[42] Altschuh D, Vix O, Rees B, Thierry J-C (1992). A conformation of Cyclosporin A in aqueous environment revealed by the X-ray structure of a Cyclosporin-Fab complex.. Science.

[43] Rini JM, Schulze-Gahmen U, Wilson IA (1992). Structural evidence for induced fit as a mechanism for antibody-antigen recognition.. Science.

[44] Schulze-Gahmen U, Rini JM, Wilson IA (1993). Detailed analysis of the free and bound conformations of an antibody. X- ray structures of Fab 17/9 and three different Fab-peptide complexes.. J Mol Biol.

[45] Chacko S, Padlan EA, Portolano S, McLachlan SM, Rapoport B (1996). Structural studies of human autoantibodies. Crystal structure of a thyroid peroxidase autoantibody Fab.. J Biol Chem.

[46] Bundle DR, Baumann H, Brisson JR, Gagne SM, Zdanov A (1994). Solution structure of a trisaccharide-antibody complex: comparison of NMR measurements with a crystal structure.. Biochemistry.

[47] Reynolds M, Fuchs A, Lindhorst TK, Perez S (2008). The hydration features of carbohydrate determinants of Lewis antigens.. Mol Simulation.

[48] Basu A, Murthy U, Rodeck U, Herlyn M, Mattes L (1987). Presence of tumor-associated antigens in epidermal growth factor receptors from different human carcinomas.. Cancer Res.

[49] Klinger M, Farhan H, Just H, Drobny H, Himmler G (2004). Antibodies directed against Lewis-Y antigen inhibit signaling of Lewis-Y modified ErbB receptors.. Cancer Res.

[50] Farhan H, Schuster C, Klinger M, Weisz E, Waxenecker G (2006). Inhibition of xenograft tumor growth and down-regulation of ErbB receptors by an antibody directed against Lewis Y antigen.. J Pharmacol Exp Ther.

[51] Johns TG, Perera RM, Vernes SC, Vitali AA, Cao DX (2007). The efficacy of epidermal growth factor receptor-specific antibodies against glioma xenografts is influenced by receptor levels, activation status, and heterodimerization.. Clin Cancer Res.

[52] Perera RM, Zoncu R, Johns TG, Pypaert M, Lee FT (2007). Internalization, intracellular trafficking, and biodistribution of monoclonal antibody 806: a novel anti-epidermal growth factor receptor antibody.. Neoplasia.

[53] Scott AM, Lee FT, Tebbutt N, Herbertson R, Gill SS (2007). A phase I clinical trial with monoclonal antibody ch806 targeting transitional state and mutant epidermal growth factor receptors.. Proc Natl Acad Sci U S A.

[54] Sundstrom M, Abrahmsen L, Antonsson P, Mehindate K, Mourad W (1996). The crystal structure of staphylococcal enterotoxin type D reveals Zn^2+^-mediated homodimerization.. Embo J.

[55] Hemmens B, Goessler W, Schmidt K, Mayer B (2000). Role of bound zinc in dimer stabilization but not enzyme activity of neuronal nitric-oxide synthase.. J Biol Chem.

[56] Ding YH, Javaherian K, Lo KM, Chopra R, Boehm T (1998). Zinc-dependent dimers observed in crystals of human endostatin.. Proc Natl Acad Sci U S A.

[57] Dunn MF (2005). Zinc-ligand interactions modulate assembly and stability of the insulin hexamer - a review.. Biometals.

[58] Calarese DA, Scanlan CN, Zwick MB, Deechongkit S, Mimura Y (2003). Antibody domain exchange is an immunological solution to carbohydrate cluster recognition.. Science.

[59] Laskowski RA, MacArthur MW, Moss DS, Thornton JM (1993). PROCHECK: a program to check the stereochemical quality of protein structures.. J Appl Cryst.

